# Molecular basis of a high Hb A_2_/Hb F*β*-thalassemia trait: a retrospective analysis, genotype-phenotype interaction, diagnostic implication, and identification of a novel interaction with *α*-globin gene triplication

**DOI:** 10.7717/peerj.15308

**Published:** 2023-05-03

**Authors:** Chayada Soontornpanawet, Kritsada Singha, Hataichanok Srivorakun, Wanicha Tepakhan, Goonnapa Fucharoen, Supan Fucharoen

**Affiliations:** 1 Centre for Research & Development of Medical Diagnostic Laboratories, Faculty of Associated Medical Sciences, Khon Kaen University, Muang, Khon Kaen, Thailand; 2 Faculty of Medicine, Mahasarakham University, Muang, Mahasarakham, Thailand; 3 Department of Pathology, Faculty of Medicine, Prince of Songkla University, Hatyai, Songkhla, Thailand

**Keywords:** β-hemoglobinopathies, β-thalassemia trait, 3.4 kb deletion β-thalassemia, High Hb F SNPs, High Hb F β-thalassemia trait

## Abstract

**Background:**

*β*^0^-thalassemia deletion removing 5´*β*-globin promoter usually presents phenotype with high hemoglobin (Hb) A_2_ and Hb F levels. We report the molecular characteristics and phenotype-genotype correlation in a large cohort of the *β*^0^-thalassemia with 3.4 kb deletion.

**Methods:**

A total of 148 subjects, including 127 heterozygotes, 20 Hb E-*β*-thalassemia patients, and a double heterozygote with *α*-globin gene triplication, were recruited. Hb and DNA analysis were performed to identify thalassemia mutations and four high Hb F single nucleotide polymorphisms (SNPs) including four base pair deletion (-AGCA) at ^A^*γ*-globin promoter, rs5006884 on OR51B6 gene, −158 ^G^*γ*-*Xmn*I, BCL11A binding motifs (TGGTCA) between 3´^A^*γ*-globin gene and 5´*δ*-globin gene.

**Results:**

It was found that heterozygous *β*^0^-thalassemia and Hb E-*β*^0^-thalassemia with 3.4 kb deletion had significantly higher Hb, hematocrit, mean corpuscular volume, mean corpuscular hemoglobin and Hb *F* values as compared with those with other mutations. Co-inheritance of heterozygous *β*^0^-thalassemia with 3.4 kb deletion and *α*-thalassemia was associated with even higher MCV and MCH values. The Hb E-*β*^0^-thalassemia patients carried a non-transfusion-dependent thalassemia phenotype with an average Hb of around 10 g/dL without blood transfusion. A hitherto undescribed double heterozygous *β*^0^-thalassemia with 3.4 kb deletion and *α*-globin gene triplication presented as a plain *β*-thalassemia trait. Most of the subjects had wild-type sequences for the four high Hb F SNPs examined. No significant difference in Hb F was observed between those of subjects with and without these SNPs. Removal of the 5´*β*-globin promoter may likely be responsible for this unusual phenotype.

**Conclusions:**

The results indicate that *β*^0^-thalassemia with 3.4 kb deletion is a mild *β*-thalassemia allele. This information should be provided at genetic counseling and prenatal thalassemia diagnosis.

## Introduction

*β*-hemoglobinopathies are inherited hemoglobin (Hb) disorders caused by mutations in the *β*-globin gene, resulting in quantitatively reduced (*β*^+^-thalassemia) or absent (*β*^0^-thalassemia) *β*-globin chain synthesis and qualitative *β*-globin chain defect called structural *β*-globin chain variant. Although some *β*-globin gene deletions have been reported, most defects are non-deletional mutants of the *β*-globin gene (Steinberg et al., 2009). In Thailand, a high prevalence of *β*-hemoglobinopathies has been described, including 20–30% of Hb E (HBB:c.79G>A) and 3–9% of *β*-thalassemia (Paiboonsukwong et al., 2022). In northeast Thailand, eight *β*-thalassemia mutations including CD 41/42 (-TTCT) (HBB:c.126_129delCTTT), CD17 (A-T) (HBB:c.52A>T), NT-28 (A-G) (HBB:c.-78A>G), CD71/72 (+A) (HBB:c.217dupA), IVSI-1 (G-T) (HBB:c.92+1G>T), IVSII-654 (C-T) (HBB:c.316-197C>T), 3.4 kb deletion and IVSI-5 (G-C) (HBB:c.92+5G>C) accounted for 97.4% of the total mutations (Yamsri et al., 2011). Generally, *β*-thalassemia heterozygote has normal or slightly decreased Hb level and reduced mean corpuscular volume (MCV) and mean corpuscular hemoglobin (MCH) with increased Hb A_2_ to around 5.0% and Hb F 0–3%. Homozygote or compound heterozygote of *β*-thalassemia with Hb E led to *β*-thalassemia major and Hb E-*β*-thalassemia diseases, with variable thalassemia phenotype. The *β*^+^-thalassemia has a milder thalassemia phenotype than the *β*^0^-thalassemia (Yamsri et al., 2011; Yamsri et al., 2015).

Interestingly, among these common mutations, it was found that the carrier of *β*-thalassemia with 3.4 kb deletion was associated with higher Hb A_2_, Hb F, MCV, and MCH values as compared with those with other mutations (Yamsri et al., 2011). A high Hb F expression may presumably lead to a milder *β*-thalassemia phenotype in homozygotic or compound heterozygotic forms. This 3.4 kb DNA deletion removes an entire *β*-globin gene, including its 5′ promoter. In this study, we have examined several genetic single nucleotide polymorphisms (SNPs) modifiers affecting Hb A_2_ and Hb F expression in a large cohort of *β*^0^-thalassemia with 3.4 kb deletion. The molecular basis, and phenotype and genotype interaction observed in both heterozygote and compound heterozygote forms, as well as a hitherto undescribed interaction of the 3.4 kb deletion *β*^0^-thalassemia with an *α*-globin gene triplication, are described.

## Materials and Methods

### Subjects and hematological analysis

Ethical approval of the study protocol was obtained from the Institutional Review Board (IRB) of Khon Kaen University, Khon Kaen, Thailand (HE632216). A total of 618 leftover specimens of *β*-thalassemia carriers were selectively recruited from our archival at the Center for Research and Development of Medical Diagnostic Laboratories (CMDL), Faculty of Associated Medical Science, Khon Kaen University and Department of Pathology, Faculty of Medicine, Prince of Songkla University, during January 2008 to December 2021. These included leftover DNA specimens of *β*-thalassemia carriers with 3.4 kb (*n* = 148), Filipino (*n* = 9), and 105 bp (*n* = 7) deletions. The 454 representative specimens of *β*^+^-thalassemia trait (*n* = 70), *β*^0^-thalassemia trait (*n* = 259), and Hb E- *β*^0^-thalassemia disease (*n* = 125) with non-deletional *β*-thalassemia mutations were used to compare with the deletional *β*^0^-thalassemia mutation. Study was done on leftover DNA specimens; consent was not required. Hematological parameters were recorded on a standard blood cell counter; Sysmex XN-9000 (Sysmex Europe SE, Norderstedt, Germany), and Hb analysis was performed using capillary electrophoresis (Capillary II Flex piercing, Sebia, France).

### Routine DNA analysis

Genomic DNA was prepared from peripheral blood leukocytes using the GF-1 Blood DNA extraction kit (Vivantis Technologies Sdn. Bhd, Selangor Darul Ehsan, Malaysia). Identification of  *α*^0^-thalassemia (SEA and THAI deletions), *α*^+^-thalassemia (3.7 kb and 4.2 kb deletions), Hb Constant Spring, Hb Paksé, *α*-globin gene triplication and common *β*-thalassemia mutations were performed using polymerase chain reaction (PCR)-based techniques (Yamsri et al., 2010; Singha et al., 2013; Chaibunruang et al., 2018; Charoenwijitkul et al., 2019). Identification of the Filipino deletion and the 105 bp deletion *β*-thalassemia were performed using gap-PCR assays as described previously (Nopparatana et al., 1999; Yamsri et al., 2012). The *Xmn* I polymorphism at −158 ^G^*γ* (C>T) (rs7482144) was determined by PCR-restriction fragment length polymorphism (PCR-RFLP) as described elsewhere (Phanrahan et al., 2019).

### DNA sequencing

**Figure 1:**
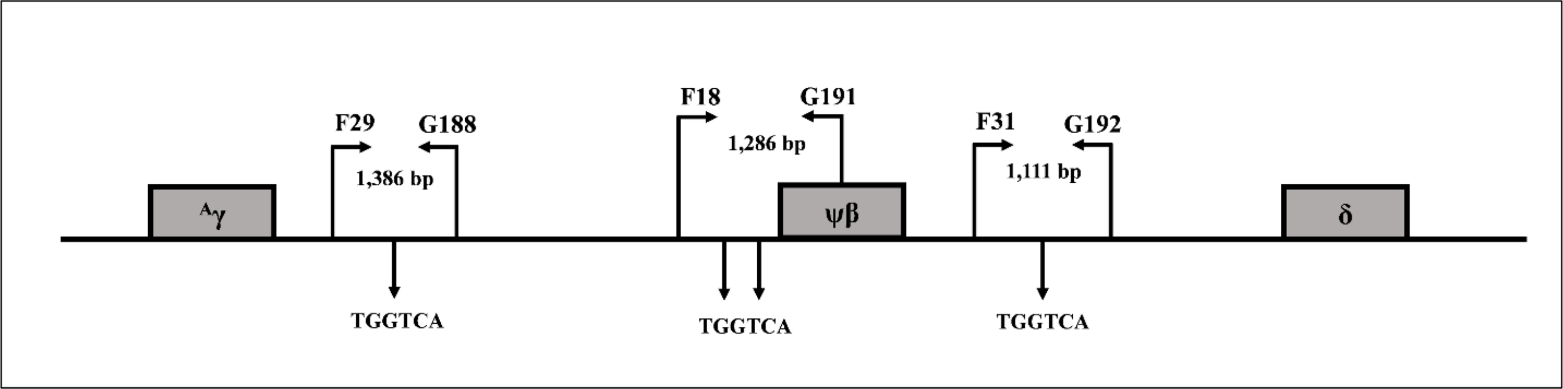
Locations and orientations of amplification primers for BCL11A. Locations and orientations of amplification primers for identification of BCL11A binding motifs (TGGTCA) by DNA sequencing within DNA sequences between +3 kb 3′  of ^*A*^*γ*-globin gene to about −1 kb 5′  of *δ*-globin gene.

Direct DNA sequencing was used to determine deletion breakpoints of the *β*^0^-thalassemia with 3.4 kb deletion generated using primers G9 (5′-TCCCCAGTTAACCTCCTATT-3′) and N1 (5′-ACATATGAGCAAGGTTGTGT-3′) on a specific fragment of 456 base pair (bp). To examine three BCL11A binding motifs (TGGTCA) located between +3 kb 3′of ^A^*γ*-globin gene and about 1 kb 5′*δ*-globin gene, the three amplified fragments using primer pairs F29 (5′-CACGAGTCAGCCTTCAGAAA-3′) & G188 (5′- GCAGAGGAGACCAGCATACA-3′) (1,386 bp), F18 (5′-GACATGGACTATTGTTCAATGA-3′) & G191 (5′-AGACAACGGGCTAATCCCTC-3′) (1,286 bp), and F31 (5′-ACCCACATT GGCATTACAC-3′) & G192 (5′-AGAATGTGTTTGTGAGGGAGGA-3′) (1,111 bp) were generated as shown in Fig. 1 and the fragments were sequenced directly. To analyze the whole *γ*-globin genes, primer F22 (5′-TACTGCGCTGAAACTGTGGC-3′) & *γ*35 (5′-AGGTAGTTGTTCCCCTTCAA-3′) and SF5 (5′-TTAACGTCTTCAGCCTACAA-3′) & SF6 (5′-CAATCTGCACACTTGAGGGC-3′) were used to amplify DNA fragments of 1,843 and 993 bp, specific for ^A^*γ*-globin gene, and primers *γ*4 (5′-GGCCTAAAACC ACAGAGA-3′) & *γ*34 (5′-AGGTAGTTGTTGTTGTTGCA-3′) and SF3 (5′-GTTTGTGTGTG TGTGAGAGC-3′) & SF4 (5′-TCTTTAGGCATGCGTCAACA-3′) were used to amplify DNA fragments of 1,787 and 1,121 bp of ^G^*γ*-globin gene, followed by DNA sequencing (Fucharoen, Shimizu & Fukumaki, 1990; Panyasai et al., 2004; Svasti et al., 2007; Singha et al., 2015) [9-12]. DNA sequencing was carried out using Sanger sequencing method on an ABI PRISM™ 3730XL analyzer (Applied Biosystems, Foster City, CA, USA).

### Identification of the four bp deletion (-AGCA) of the^***A***^*γ*-globin gene promoter

**Figure 2:**
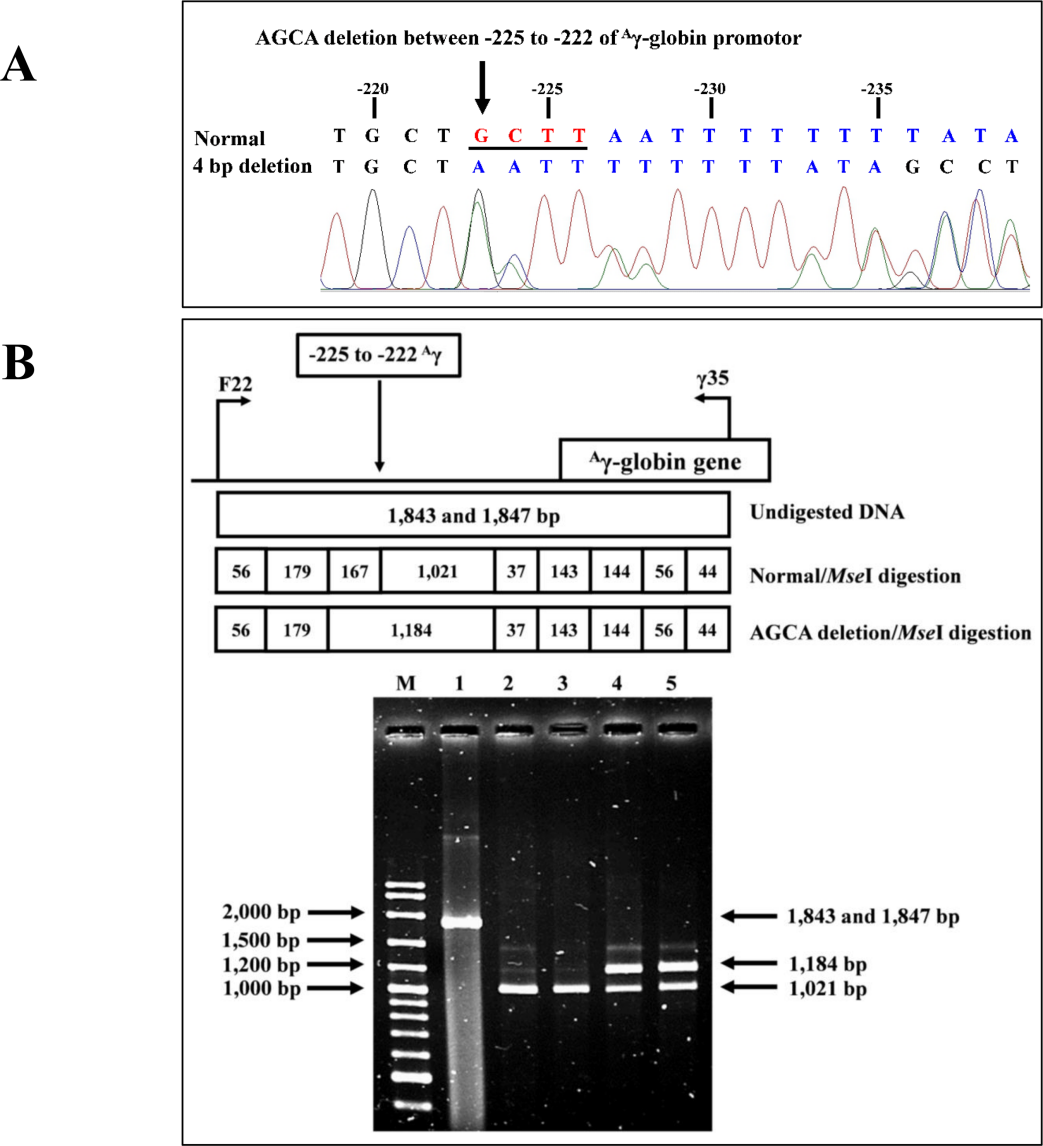
DNA sequencing profile and identification of four bp (-AGCA) deletion in the A-gamma globin gene. (A) DNA sequencing profile of a representative subject with heterozygosity for the four bp deletion (-AGCA) between −225 to −222 of ^*A*^*γ*-globin gene. (B) Identification of four bp deletion (-AGCA) between −225 to −222 of ^*A*^*γ*-globin gene using PCR-RFLP by *Mse* I digestion. Locations and orientations of primers F22 and *γ*35 are indicated to produce PCR fragment of 1,843 bp or 1,847 bp. The *Mse* I-digested fragments of 1,021 bp and 167 bp for wide-type allele and 1,184 bp for mutant allele are depicted. M represents the GeneRular 100 bp Plus DNA ladder. Lane 1: undigested amplified DNA, lanes 2 & 3: *Mse* I-digested amplified DNA of homozygous wide-type allele, and lanes 4 & 5: *Mse* I-digested amplified DNA of heterozygous for the four bp deletion (-AGCA) of ^*A*^*γ*-globin gene.

The PCR-RFLP assay was developed to detect four bp deletion (-AGCA) at −225 to −222 of the ^A^*γ*-globin gene promoter (rs1451276553). Specific amplification of ^A^*γ*-globin gene promoter was performed using primers F22 and *γ*35 to produce a fragment of 1,847 bp wild-type or 1,843 bp specific for the four bp deletion. This was confirmed by DNA sequencing and PCR-RFLP assay. The PCR product was digested with a *Mse* I restriction enzyme (5′-T^▾^TAA-3′) (Vivantis Technologies Sdn. Bhd, Selangor Darul Ehsan, Malaysia). After digestion, the 1,021 bp and 167 bp indicate wild type, whereas the 1,184 bp is a specific fragment for the AGCA deletion, as shown in Fig. 2.

### Allele-specific PCR assay for identification of rs5006884 polymorphism in the OR51B6 gene

**Figure 3:**
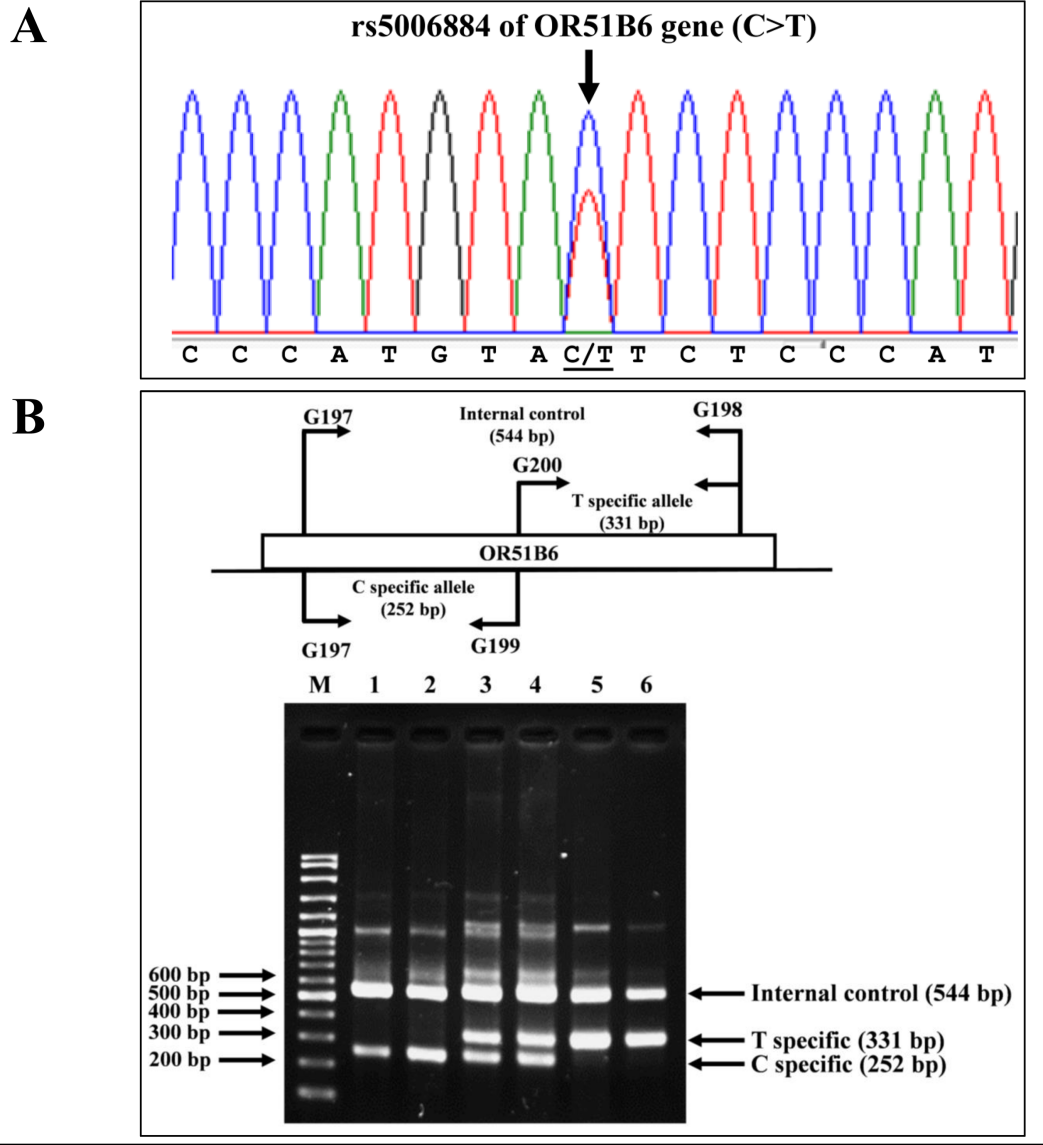
DNA sequencing profile and identification of the rs5006884 on OR51B6. (A) DNA sequencing profile of a representative subject with heterozygosity for the rs5006884 on OR51B6 gene. (B) Characterization of the rs5006884 on OR51B6 gene by allele-specific PCR. The locations and orientations of primers (G197 & G198), (G200 & G198), and (G197 & G199) are indicated to produce fragments of 544 bp, 331 bp, and 252 bp specific for internal control, T-specific allele (mutant) and C-specific allele (wide-type) of rs5006884, respectively. M represents the GeneRular 100 bp Plus DNA ladder. Lanes 1 & 2: homozygous wide-type allele (C/C), lanes 3 & 4: heterozygous for C/T alleles, and lanes 5 & 6: homozygous for T/T alleles.

Allele-specific PCR was developed to identify the rs5006884 (NC_000011.10:g.5352021C>T) in the OR51B6 gene on chromosome 11. In this system, primers G197 (5′-AAGACCAGG ATGCAGCAGAT-3′) and G198 (5′-ACCTCGGAGTGACATTGACC-3′) were used to produce an amplified fragment of 544 bp internal control. Specific amplification of the C allele (252 bp) and T allele (331 bp) were obtained using primers G197 & G199 (5′-CCTACTGTCGATCCCATGTAC-3′), and G200 (5′-AGACAGAAAGCATGGGAGAA-3′) & G198, respectively. The PCR reaction mixture (50 µL) contains 50–200 ng of genomic DNA, 200 µM of dNTPs, 30 pmol of each primer, and 1 unit of *Taq* DNA polymerase (New England Biolab, Inc., USA) in 10 mM Tris–HCl buffer pH 8.3, 50 mM KCl, 3 mM MgCl_2_, and 0.01% gelatin. The PCR consists of an initial denaturation at 94 °C for 3 min followed by 30 cycles of amplification at (94 °C for 1 min and 65 °C for 90 s). The PCR product was analyzed on 2% agarose gel electrophoresis and visualized under UV light after ethidium bromide staining. Confirmation of SNP rs5006884 was done using DNA sequencing (Fig. 3).

### Statistical analysis

Hematological parameters of the subjects were presented as descriptive statistics, mean and standard deviation. The Mann–Whitney *U* test or Wilcoxon test was performed to compare variables between two dependent groups with non-normal distribution using the R software (version 4.1.0) (The R Foundation, Vienna, Austria). Statistical significance was set at *P*-value <0.05.

## Results

**Figure 4:**
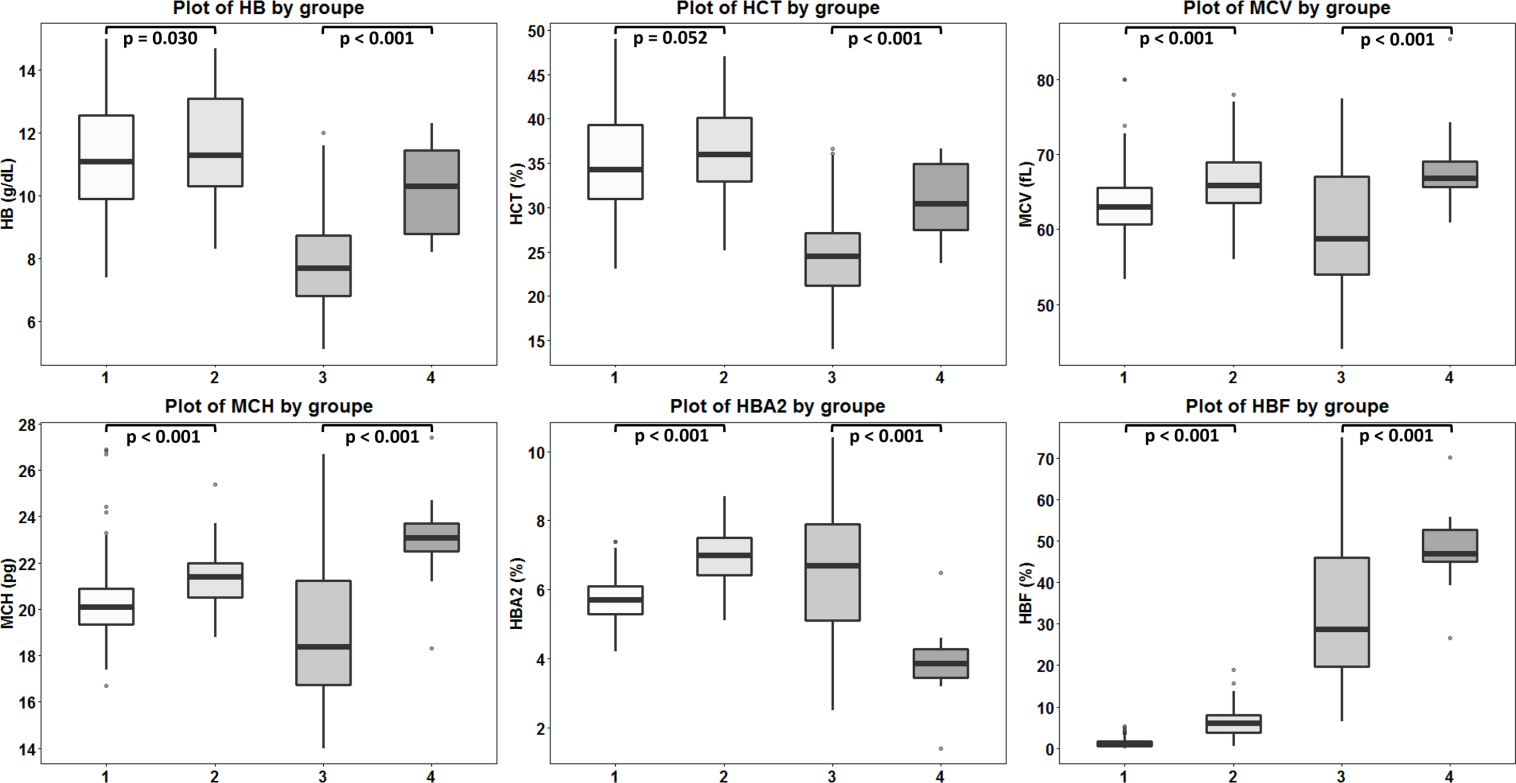
Comparison of hematological values of the four groups of *β* -thalassemia. Box plots demonstrating medians and interquartile ranges in comparison of Hb, Hct, MCV, MCH, Hb A_2_ and Hb F values of four *β*-thalassemia genotypes including (1): *β*^0^-thalassemia trait with point mutations (*n* = 259), (2): *β*^0^-thalassemia trait with 3.4 kb deletion (*n* = 103), (3): Hb E- *β*^0^-thalassemia with point mutations (*n* = 125) and (4): Hb E- *β*^0^-thalassemia with 3.4 deletion (*n* = 20).

DNA sequencing of the deletion breakpoint of the *β*^0^-thalassemia with 3.4 kb deletion encountered in Thai patients identified the deletion as NG_000007.3:g.69825_73314del3488 or NC_000011.10:g.5224302_5227791del3490 (GenBank Accession no. OQ161123), exactly the same with that reported previously in a Chinese patient (He et al., 2018). Table 1 and Fig. 4 demonstrated the hematological parameters associated with the *β*^0^-thalassemia trait with 3.4 kb deletion (*n* = 103) compared to those of the *β*^0^-thalassemia trait with other mutations (*n* = 259) encountered in our series. As shown in the table, significant higher Hb (11.6 ± 1.7 *vs.* 11.2 ± 1.6 g/dL), hematocrit (Hct) (36.1 ± 5.2 *vs.* 34.8 ± 5.2%), MCV (66.3 ± 4.3 *vs.* 63.2 ± 3.8 fL), MCH (21.3 ± 1.3 *vs.* 20.2 ± 1.4 pg), Hb A_2_ (6.9 ± 0.8 *vs.* 5.7 ± 0.6%) and Hb F (6.5 ± 3.4 *vs.* 1.3 ± 0.9%) values were observed for the former group of subjects. These are also the cases for the patients with compound heterozygous *β*^0^-thalassemia and Hb E. We observed significant higher Hb, Hct, MCV, MCH, and Hb F (48.1 ± 8.3 *vs.* 32.2 ± 15.6%) but lower Hb A_2_ (3.9 ± 1.1 *vs.* 6.5 ± 1.9%) values for the patients with 3.4 kb deletion (*n* = 20) as compared to those with other *β*^0^-thalassemia mutations (*n* = 125). These results indicate that the 3.4 kb deletion *β*^0^-thalassemia is associated with high Hb A_2_ and Hb F in a heterozygote state and a mild hematological phenotype when found in association with Hb E in a Hb E- *β*^0^-thalassemia disease. The effect of co-inheritance of *α*-hemoglobinopathy on the hematological phenotype of heterozygous *β*-thalassemia with 3.4 kb deletion was summarized in Table 2. Significant elevation of MCV and MCH values and a reduction of Hb F (3.0 ± 3.3%) were observed in heterozygous *β*-thalassemia with 3.4 kb deletion with heterozygous *α*^+^-thalassemia (– *α*/*αα*, *n* = 13). Lower Hb F but not Hb A_2_ were also observed in double heterozygotes for 3.4 kb deletion *β*-thalassemia and other deletional forms of *α*-thalassemia including homozygous *α*^+^-thalassemia (– *α*/– *α*, *n* = 1), *α*^o^-thalassemia (--/ *αα*, *n* = 2) and Hb H disease (--/- *α*, *n* = 1). In contrast, we observed that co-inheritance of a non-deletional *α*-thalassemia, the Hb Constant Spring (*α*^CS^*α*/*αα*, *n* = 7), did not lead to a reduction in Hb F expression, although it did have some hematological improvement. However, no statistical analysis was performed due to the small sample sizes of these *α*-hemoglobinopathies.

**Table 1:** Hematological parameters and Hb analysis of subjects with *β*^0^-thalassemia (3.4 kb deletion) as compared with corresponding genotypes of *β*^0^-thalassemia with other mutations. Values are presented as mean ± standard variation. Statistical analysis was done using the Mann–Whitney *U*-test. M, Male, F, Female.

**Group**	*β*-genotype	N	Sex (M/F)	RBC (10 ^**12**^/L)	Hb (g/dL)	Hct (%)	MCV (fL)	MCH (pg)	MCHC (g/dL)	RDW (%)	Hbs E+A_**2**_ (%)	Hb A_**2**_ (%)	Hb F **(%)**
Pure *β*^0^-thalassemia trait ^a^	*β*^0^/*β*^A^	259	119/140	5.5 ± 0.9	11.2 ± 1.6	34.8 ± 5.2	63.2 ± 3.8	20.2 ± 1.4	32.2 ± 1.8	17.1 ± 1.8	–	5.7 ± 0.6	1.3 ± 0.9
Pure *β*^3.4^-thalassemia trait	*β*^3.4^/*β*^A^	103	55/48	5.5 ± 0.8	11.6 ± 1.7	36.1 ± 5.2	66.3 ± 4.3	21.3 ± 1.3	32.2 ± 1.3	19.2 ± 2.0	–	6.9 ± 0.8	6.5 ± 3.4
*P*-value				0.50	0.030^*^	0.052	<0.001^*^	<0.001^*^	0.099	<0.001^*^	–	<0.001^*^	<0.001^*^
*β*^0^-thalassemia/Hb E ^b^	*β*^0^/*β*^E^	125	60/65	4.1 ± 1.1	7.8 ± 1.5	24.8 ± 4.9	60.4 ± 8.6	19.0 ± 2.9	31.7 ± 1.9	29.5 ± 3.9	63.2 ± 16.2	6.5 ± 1.9	32.2 ± 15.6
*β*^3.4^-thalassemia/Hb E	*β*^3.4^/*β*^E^	20	8/12	4.4 ± 0.7	10.2 ± 1.4	30.5 ± 4.6	68.3 ± 5.7	23.0 ± 2.1	33.4 ± 1.8	22.4 ± 1.7	48.4 ± 6.3	3.9 ± 1.1	48.1 ± 8.3
*P*-value				0.150	<0.001^*^	<0.001^*^	<0.001^*^	<0.001^*^	0.009^*^	<0.001^*^	<0.001^*^	<0.001^*^	<0.001^*^

**Notes.**

a including *β*^41/42(−TTCT)^(*n* = 148), *β*^17(A>T)^(*n* = 92), *β*^71/72(+A)^ (*n* = 12), and *β*^IV SI−1(G>T)^ (*n* = 7).

b including *β*^41/42(−TTCT)^(*n* = 80), *β*^17(A>T)^(*n* = 33), *β*^71/72(+A)^ (*n* = 7), and *β*^IV SI−1(G>T)^ (*n* = 5).

* Significant difference at *P*-value <0.05 using Mann–Whitney *U*-test.

Interestingly, an adult male patient was encountered with a double heterozygote for *β*-thalassemia with 3.4 kb deletion and an *α*-globin gene triplication (*ααα*^anti^^−^^3^^.^^7^/ *αα*), a hitherto undescribed condition. He had relatively normal Hb and Hct values with reduced MCV & MCH characteristics of a plain *β*-thalassemia carrier. Elevated Hb A_2_ (7.2%) and Hb F (6.8%) were observed. The result indicates that, unlike other *β*^0^-thalassemia alleles, the interaction of *α*-globin gene triplication (*ααα*^anti^^−^^3.7^/ *αα*) and a *β*^0^-thalassemia with 3.4 kb deletion does not lead to a *β*-thalassemia disease in heterozygotic form.

SNPs were examined in representative subjects to determine if the existence of known Hb F associated SNPs are responsible for the high Hb A_2_ and Hb F phenotype in our patients. As shown in Table 3, the majority of the subjects had wild-type sequences for the three SNPs, including deletion of four nucleotides (-AGCA) between −225 to −222 on ^A^*γ*-globin promotor, rs5006884 (C>T) of the OR51B6 gene, and ^G^*γ*-*Xmn* I of ^G^*γ*-globin gene promoter. In addition, no significant difference in Hb F and other hematological parameters was observed between those of subjects with and without these SNPs. DNA sequencing of the three BCL11A binding motifs (TGGTCA) between the 3′ ^A^*γ*-globin gene and the 5′ *δ*-globin gene (Fig. 1) identified no alteration of the nucleotide at the binding motifs and their vicinities. Further sequencing of the whole ^A^*γ*- and ^G^*γ*-globin genes in representative subjects identified no mutation.

## Discussion

The carrier of *β*-thalassemia usually accompanies elevated Hb A_2_ and normal Hb F levels. Hb A_2_ isa product of tetrameric assembly of two *α*- and two *δ*-globin chains (*α*_2_*δ*_2_), which is synthesized at a low level (2.5−3.5%) in normal adult individuals. Several genetic and non-genetic conditions are associated with Hb A_2_ expression (Singha et al., 2021). Among many different *β*-thalassemia genes in Thailand, we observed that only the 3.4 kb deletion *β*^0^-thalassemia is always associated in heterozygotic form with high Hb A_2_ (6.9 ± 0.8%) and Hb F (6.5 ± 3.4%) levels. Heterozygosity for other *β*-thalassemia alleles is usually found with elevated Hb A_2_ (5.7 ± 0.6%) but not Hb F (1.3 ± 0.9%) (Yamsri et al., 2010; Yamsri et al., 2011; Yamsri et al., 2015; Yamsri et al., 2022). Hematological characteristics in a large cohort of *β*-thalassemia carriers with this mutation listed in Table 1 confirmed this phenotype characteristic. This 3.4 kb deletion *β*^0^-thalassemia has been frequently described among Southeast Asian and Chinese patients. It has rarely been encountered on population of India, Pakistan and Bangladesh (Nadkarni et al., 2019; Khaliq, 2022; Chowdhury, Sultana & Das, 2022). This 3,488 bp deletion *β*-thalassemia allele removes DNA between positions −125 to +78 of the *β*-globin gene, eliminating the CACCC, CCAAT, and TATA elements in the *β*-globin gene promoter. The high Hb F characteristic of this *β*^0^-thalassemia allele could likely ameliorate the patient’s clinical phenotype. As shown in Table 1, when found in combination with Hb E, it could lead to non-transfusion-dependent thalassemia (NTDT). As compared to other *β*^0^-thalassemia/Hb E, which is generally associated with severe transfusion-dependent thalassemia (TDT), the patients with *β*^3^^.^^4^-thalassemia/Hb E had milder NTDT phenotype with significantly higher Hb, Hct, MCV, MCH, MCHC, and Hb F but lower RDW values. We have also documented recently that the patients with homozygous *β*^0^-thalassemia of 3.4 kb deletion were presented with mild NTDT phenotype with Hb ranging from 7–9 g/dL and did not require regular blood transfusion (Soontornpanawet, Singha & Fucharoen, 2022). It is noteworthy for a compound Hb E- *β*-thalassemia with 3.4 kb deletion that the patient had significant higher Hb, Hct, MCV, MCH, and Hb F (48.1 ± 8.3 *vs.* 32.2 ± 15.6%) but lower Hb A_2_ (3.9 ± 1.1 *vs.* 6.5 ± 1.9%) as compared to those of other *β*^0^-thalassemia mutations (Table 1). This lower Hb A_2_ in Hb E-*β*^0^-thalassemia with 3.4 kb deletion as compared with Hb E-*β*^0^-thalassemia with non-deletional mutations is most likely due to the higher Hb F expression. There is an inverse relationship between Hb A_2_ and Hb F expression in *β*-thalassemia. Red blood cells that produce relatively large amount of Hb F will produce less Hb A_2_, and vice versa (Singha et al., 2018). It is conceivable that although the 3.4 kb deletion results in *β*^0^-thalassemia, the increase in Hb F may compensate for the complete absence of Hb A and ameliorate the severity of the disease as seen in our patients.

**Table 2:** Hematological characteristics of heterozygous *β*^0^-thalassemia with 3.4 kb deletion with and without *α*-hemoglobinopathy including a novel genetic interaction of *ααα*^anti−3.7^ and *β*^0^-thalassemia with 3.4 kb deletion. Values are presented as mean ± standard variation or as raw data where appropriate. Statistical analysis was done using the Mann–Whitney U-test. M, Male; F, Female.

*α*-genotype	No	Sex (M/F)	RBC (10^12^/L)	Hb (g/dL)	Hct (%)	MCV (fL)	MCH (pg)	MCHC (g/dL)	RDW (%)	Hb A_2_ (%)	Hb F (%)
*αα*/*αα*	103	55/48	5.5 ± 0.8	11.6 ± 1.7	36.1 ± 5.2	66.3 ± 4.3	21.3 ± 1.3	32.2 ± 1.3	19.2 ± 2.0	6.9 ± 0.8	6.5 ± 3.4
- *α*/*αα*	13	10/3	5.2 ± 1.1	12.5 ± 1.9	38.7 ± 5.2	72.3 ± 6.5^a^	22.9 ± 1.7^b^	31.6 ± 1.7	15.3 ± 1.9^c^	6.9 ± 0.7	3.0 ± 3.3^d^
*α*^CS^*α*/*αα*	7	2/5	5.4 ± 0.8	12.0 ± 1.4	36.6 ± 4.6	68.3 ± 3.9	22.3 ± 1.6	32.7 ± 0.8	17.9 ± 2.5	6.8 ± 0.8	9.1 ± 5.6
- *α*/- *α*	1	1/0	na	na	na	71.4	na	na	na	7.2	na
-- /*αα*	2	0/2	4.5	10.3	30.7	67.8	22.7	33.6	18.1	6.3, 7.0	3.9, 3.6
-- /- *α*	1	0/1	5.3	9.2	29.7	51.0	15.8	31.2	Na	5.0	2.5
*ααα* ^*anti*−3.7^ **/*αα***	**1**	**1/0**	**6.4**	**13.0**	**40.0**	**62.0**	**20.3**	**32.5**	**13.7**	**7.2**	**6.8**

**Notes.**

na not available CS Hb Constant Spring

a Significant difference of MCV between *αα*/*αα* and - *α*/*αα* genotype at *p*-value = 0.006.

b Significant difference of MCH between *αα*/*αα* and - *α*/*αα* genotype at *p*-value = 0.049.

c Significant difference of RDW between *αα*/*αα* and - *α*/*αα* genotype at *p*-value = 0.014.

d Significant difference of Hb F between *αα*/ *αα* and - *α*/*αα* genotype at *p*-value = 0.001.

**Table 3:** Hematological comparison of *β*^0^-thalassemia trait with 3.4 kb deletion according to the three SNPs including a 4 bp deletion (-AGCA) in ^A^*γ*-globin promoter, rs5006884 (C>T) of the OR51B6 gene, and ^G^*γ-Xmn* I in ^G^*γ*-globin promoter. Values are presented as mean ± standard variation or as raw data where appropriate. Statistical analysis was done using the Mann–Whitney U-test.

**Genotype**	**N**	**Sex** **(M/F)**	**RBC** **(10** ^**12**^ **/L)**	**Hb** **(g/dL)**	**Hct** **(%)**	**MCV** **(fL)**	**MCH** **(pg)**	**MCHC** **(g/dL)**	**RDW** **(%)**	**Hb A**_**2**_ **(%)**	**Hb F** **(%)**
**Four bp deletion (-AGCA) in** ^**A**^ *γ*-globin promoter ^*^											
WT/WT	83	43/40	5.5 ± 0.8	11.7 ± 1.7	36.1 ± 5.3	65.8 ± 4.0	21.2 ± 1.3	32.3 ± 1.2	19.3 ± 2.0	7.0 ± 0.7	6.3 ± 3.2
WT/-4 bp	1	1/0	6.2	13.2	39.7	64.0	21.4	33.2	21.7	7.0	6.8
**rs5006884 (C>T) /** **OR51B6 gene**											
C/C	68	36/32	5.5 ± 0.8	11.6 ± 1.7	35.8 ± 5.1	65.8 ± 4.1	21.2 ± 1.3	32.3 ± 1.2	19.3 ± 1.9	6.9 ± 0.7	6.4 ± 3.7
C/T	13	5/8	5.2 ± 0.8	11.0 ± 1.6	34.1 ± 5.0	66.0 ± 4.0	21.4 ± 1.1	32.4 ± 1.3	19.6 ± 1.3	7.3 ± 0.9	7.3 ± 2.1
*P*-value			0.280	0.346	0.315	0.823	0.813	0.993	0.987	0.126	0.238
^**G**^ *γ* ***-Xmn*** **I in G*γ*-globin promoter** ^*^											
C/C	52	26/26	5.5 ± 0.8	11.8 ± 1.7	37.0 ± 5.5	67.4 ± 4.2	21.5 ± 1.2	31.5 ± 2.5	18.4 ± 2.1	6.9 ± 0.9	6.6 ± 3.9
C/T	3	2/1	5.3, na	10.3, na	40.2, 34.2	69.0, 64.4	19.3, na	30.0, na	22.6, na	6.7 ± 0.7	5.0 ± 5.7

**Notes.**

na, not available.

* Statistical analysis was not performed due to small sample size.

As shown in Table 2, co-inheritance of the 3.4 kb deletion *β*^0^-thalassemia and *α*-thalassemia (-*α*/*αα*, *α*^CS^*α*/*αα*, -*α*/-*α*, --/*αα*) could further improve the hematological phenotype of the patients due to the balance between *α*- and non-*α*-globin chains. Fortunately, in all cases of these double heterozygotes, the Hb A_2_ levels are still within the diagnostic range for a *β*-thalassemia carrier. Interestingly, we have encountered a subject with a hitherto undescribed condition of double heterozygote for the *β*^3.4kb deletion^ and *α*-globin gene triplication (*ααα*^anti^^−^^3^^.^^7^/*αα*). This subject presented with *β*-thalassemia trait phenotype without anemia with Hb 13.0 g/dL and Hct 40.0%, Hb A_2_ 7.2%, and Hb F 6.8% (Table 2). The heterozygous *β*-thalassemia with *α*-globin gene triplication generally presents with thalassemia intermedia phenotype due to more excess *α*-globin chain and imbalance in *α*- and *β*-globin chain synthesis (Steinberg et al., 2009). This might be explained by the fact that, unlike other point mutations, the *β*^3.4kb deletion^ is associated with increased expression of both *δ*- and *γ*-globin genes and could therefore improve the balance between *α*- and non- *α*-globin chains. This information indirectly supports that the *β*^0^-thalassemia with 3.4 kb deletion is a mild *β*^0^-thalassemia allele. We observed that this high Hb A_2_ and Hb F *β*-thalassemia trait is also the case with the Filipino *β*-thalassemia deletion occasionally encountered in our setting, although with different extent of Hb F expression. This Filipino *β*-thalassemia deletion [NG_000007.3:g.66258_184734del118477] was described originally as approximately 45 kb long starting from position -4,279 bp relative to the mRNA cap site of the *β*-globin gene but with an uncertain 3′ breakpoint. It was later described using gap-PCR and DNA sequencing as 118 kb in length, with the 5′ breakpoint at position −4,279 of mRNA cap site, and the 3′ breakpoint extending to the downstream olfactory receptor (OR) region where it deletes four functional OR genes and two OR pseudogenes including the OR52A1 that contains a *γ*-globin gene enhancer (Yamsri et al., 2012; Teh et al., 2018; Yasin et al., 2022). Table 4 compared the hematological parameters of *β*^0^-thalassemia carriers with three different *β*-thalassemia deletions in our series, including the 3.4 kb deletion (*n* = 103), the Filipino *β*^0^-thalassemia (*n* = 9), and 105 bp *β*^0^-thalassemia deletion (*n* = 7), all without *α*-thalassemia. The 3.4 kb deletion and the Filipino *β*^0^-thalassemia are associated with high Hb A_2_ and high Hb F *β*-thalassemia trait but not the 105 bp *β*^0^-thalassemia. In fact, this is not unexpected. The 105 *β*^0^-thalassemia is caused by a DNA deletion of −24 or −25 to +80 or +81 relative to the cap site of *β*-globin mRNA. Unlike the 3.4 kb deletion and the Filipino *β*^0^-thalassemia with 118 kb deletion, this 105 bp deletion does not remove the *β*-globin gene promoter TATA, CCAAT, and CACCC motifs (Nopparatana et al., 1999).

**Table 4:** Hematological parameters of *β*^0^-thalassemia carriers with 3.4 kb deletion (*β*^3.4^/*β*^A^, *n* = 103), 118 kb Filipino deletion (*β*^Fil^/*β*^A^, *n* = 9) and 105 bp deletion (*β*^105^/*β*^A^, *n* = 7) as compared to those of *β*^0^-thalassemia with other mutations (*β*^0^/*β*^A^, *n* = 259) and -28 A>G *β*^+^-thalassemia (*β*^+^/*β*^A^, *n* = 70) in our series. Values are presented as mean ± standard variation. Statistical analysis was done using the Mann–Whitney U-test. M, Male; F, Female.

*β*-genotype	*α*-genotype	No.	Sex (M/F)	RBC (10^6^/ µL)	Hb (g/dL)	HCT (%)	MCV (fL)	MCH (pg)	MCHC (g/dL)	RDW (%)	Hb A_2_ (%)	Hb F (%)
*β*^0^/*β*^A^	*αα*/*αα*	259	119/140	5.5 ± 0.9	11.2 ± 1.6	34.8 ± 5.2	63.2 ± 3.8	20.2 ± 1.4	32.2 ± 1.8	17.1 ± 1.8	5.7 ± 0.6	1.3 ± 0.9
*β*^+^/*β*^A^	*αα*/*αα*	70	40/30	5.5 ± 0.8	12.6 ± 1.8	38.6 ± 5.2	70.5 ± 4.4	23.3 ± 2.1	32.6 ± 1.8	16.7 ± 1.9	5.9 ± 0.6^a^	1.4 ± 0.8
*β*^3.4^/*β*^A^	*αα*/*αα*	103	55/48	5.5 ± 0.8	11.6 ± 1.7	36.1 ± 5.2	66.3 ± 4.3	21.3 ± 1.3	32.2 ± 1.3	19.2 ± 2.0	6.9 ± 0.8^b^,^c^	6.5 ± 3.4^b^,^c^
*β*^Fil^/*β*^A^	*αα*/*αα*	9	5/4	5.1 ± 0.6	11.7 ± 1.3	37.1 ± 3.9	69.2 ± 5.4	23.1 ± 4.0	32.0 ± 0.7	16.8 ± 1.3	6.4 ± 0.6^d^,^e^	4.0 ± 3.2^f^,^g^
*β*^105^/*β*^A^	*αα*/*αα*	7	2/5	5.1 ± 0.5	10.6 ± 0.9	32.9 ± 2.7	63.6 ± 3.4	20.6 ± 1.3	32.1 ± 0.4	16.6 ± 1.0	5.3 ± 0.7^h^	1.4 ± 1.1

**Notes.**

a Significant difference of Hb A_2_ between *β*^0^/ *β*^A^ and *β*^+^/*β*^A^ genotype at *p*-value = 0.009.

b Significant difference of Hb A_2_ and Hb F between *β*^0^/*β*^A^ and *β*^3.4^/ *β*^A^ genotype at *p*-value <0.001.

c Significant difference of Hb A_2_ and Hb F between *β*^+^/*β*^A^ and *β*^3.4^/*β*^A^ genotype at *p*-value <0.001.

d Significant difference of Hb A_2_ between *β*^0^/ *β*^A^ and *β*^Fil^/*β*^A^ genotype at *p*-value = 0.002.

e Significant difference of Hb A_2_ between *β*^+^/ *β*^A^ and *β*^Fil^/*β*^A^ genotype at *p*-value = 0.012.

f Significant difference of Hb F between *β*^0^/ *β*^A^ and *β*^Fil^/*β*^A^ genotype at *p*-value <0.001.

g Significant difference of Hb F between *β*^+^/*β*^A^ and *β*^Fil^/*β*^A^ genotype at *p*-value = 0.002.

h Significant difference of Hb A_2_ between *β*^+^/ *β*^A^ and *β*^105^/*β*^A^ genotype at *p*-value = 0.022.

It has been noted that variation preliminarily in the two SNPs, rs4895441 and rs9399137 in HBS1L-MYB intergenic region, may have a small effect on Hb F expression in this *β*-thalassemia allele (Tepakhan, Kanjanaopas & Srewaradachpisal, 2020). We have also documented that the G allele of rs4895441 and C allele of rs9399137, alone or in combination with other SNPs, could explain the mild phenotypic expression of NTDT associated with Hb E-*β*-thalassemia disease (Phanrahan et al., 2019). To understand the high Hb F characteristic of the 3.4 kb deletion *β*^0^-thalassemia allele, we have examined other SNPs associated with high Hb A_2_ & Hb F expression. These included the -AGCA deletion at −225 to −222 of ^A^*γ*-globin gene promoter, rs5006884 (C>T) on OR51B6 gene, −158^G^*γ*-*Xmn* I, BCL11A binding motifs between ^A^*γ*- and *δ*-globin genes as well as the whole *γ*-globin gene sequencing. It has been shown that the four bp deletion (-AGCA) at −225 to −222 of the ^A^*γ*-globin gene was associated with reduced ^A^*γ*-globin chain and elevation of ^G^*γ* chain with slightly increased Hb F level in *β*-thalassemia subjects (Manca et al., 1991; Ugrin et al., 2016). The rs5006884 (C>T) on OR51B6 gene located upstream of *β*-globin gene cluster might intervene in the tetramer formation of *α*-globin chain with *β*- and *δ*-globin chains and regulated Hb A_2_ level in *β*-thalassemia carriers (Cyrus et al., 2019). The −158^G^*γ*-*Xmn* I polymorphism is associated with high Hb F expression in *β*-hemoglobinopathies during erythropoietic stress (Thein, 2017). This ^G^*γ*-*Xmn* I is almost linked to the +25 (G>A)^A^*γ*-globin promoter (rs368698783). The polymorphism leads to a decreased Ly-1 antibody reactive clone (LYAR) binding efficiency, which is the repressor of *γ*-globin genes (Bianchi et al., 2016; Chen et al., 2017). BCL11A maintains silencing of *γ*-globin expression in adult erythroid cells and direct promoter repression to control the fetal to adult Hb switch in humans. The BCL11A binding motifs (TGGTCA or TGACCA) have been identified on the *β*-globin gene cluster at the third hypersensitive site (HS3) of the *β*-LCR, downstream of ^A^*γ*-globin gene and upstream of *δ*-globin gene (Sankaran et al., 2008; Sankaran, Xu & Orkin, 2010; Liu et al., 2018). Mutation in these binding motifs may therefore result in increased Hb F expression. As shown in Table 3, we did not observe the association of these SNPs with high Hb F and higher hematological values for the *β*^0^-thalassemia with 3.4 kb deletion. In fact, most of the cases had wild-type SNPs sequences, and no mutation was found in the BCL11A binding motifs between 3′^A^*γ*-globin gene and 5′ *δ*-globin gene and within ^A^*γ*- and ^G^*γ*-globin genes of the patients examined. It is, therefore, unlikely that the unusually high Hb A_2_ and Hb F *β*-thalassemia trait of the *β*^0^-thalassemia with 3.4 kb deletion is caused by the effect of these high Hb F SNPs. However, this might be explained by the fact that removal of the 5′  *β*-globin promoter in the 3.4 kb deletion may release competition of the *β*-globin gene for the upstream locus control region (*β*-LCR), leading to its increased interaction with the *γ*- and *δ*-globin genes *in cis*, increasing their expression resulting in elevation of Hb F and Hb A_2_ (Thein, 2018). Recently, it has been documented that disrupting the adult *β*-globin promoter reactivates *γ*-globin gene expression by promoter competition model in the Hb switching (Topfer et al., 2022). The 3.4 kb deletion removing *β*-globin promoter and the entire *β*-globin gene is therefore associated with elevated Hb F in addition to elevated Hb A_2_ in heterozygotic form.

## Conclusions

Nonetheless, the high Hb, Hct, MCV, MCH, Hb A_2_, and Hb F values in the heterozygote state and a mild hematological phenotype observed in Hb E-*β*^3.4kb deletion^ disease and double heterozygosity for *α*-globin gene triplication (*ααα*^anti^^−3.7^) and *β*^3.4kb deletion^ support that the *β*^3.4kb deletion^ is mild *β*^0^-thalassemia allele and might ameliorate severity when occurring in homozygote or compound heterozygote state. This high Hb A_2_ and high Hb F *β*-thalassemia characteristic could also be a useful marker for the *β*^3.4kb deletion^ before being further confirmed by PCR analysis. This information related to the 3.4 kb deletion *β*-thalassemia should be provided to the patient’s family for the appropriate management and genetic counseling. This is especially important information for routine prenatal thalassemia screening in a prevention and control program in the regions.

##  Supplemental Information

10.7717/peerj.15308/supp-1 Supplemental Information 1 Raw hematological data for tables and figures

10.7717/peerj.15308/supp-2 Supplemental Information 2 Genbank OQ161123

## Additional Information and Declarations

### Data Availability

The following information was supplied regarding data availability: The hematological data is available in the Supplementary File. The sequence is available at GenBank: OQ161123.
