# Supplementary material for: Molecular basis of a high Hb A2/Hb Fβ-thalassemia trait: a retrospective analysis, genotype-phenotype interaction, diagnostic implication, and identification of a novel interaction with α-globin gene triplication
Source: PeerJ. 2023 May 3;11:e15308. doi: 10.7717/peerj.15308 (PMC10163868; doi:10.7717/peerj.15308)
Supplement: Supplemental Information 2 [file peerj-11-15308-s002.pdf]

Kritsada Singha <kritsada.si@msu.ac.th>

GenBank OQ161123

gb-admin@ncbi.nlm.nih.gov <gb-admin@ncbi.nlm.nih.gov> 5 มกราคม 2566 เวลา 22:17
ถึง: kritsada.si@msu.ac.th

Dear GenBank Submitter:

Based on the data submitted to us, the scheduled release date for your submission is:

Jan 10, 2023

If this date is not correct, please let us know as soon as possible, otherwise this submission will be released on the date indicated above.

Changes may have been made to your original submission in order to conform to database annotation conventions. See the following for list of possible modifications: https://ncbi.nlm.nih.gov/genbank/flatfile_changes/

If you need to revise your record(s), follow these directions to format your update request: https://www.ncbi.nlm.nih.gov/Genbank/update.html

Send properly formatted updates to: gb-admin@ncbi.nlm.nih.gov
Do not make a new submission for an update request.

Thank you for your submission, a contribution which will benefit the scientific community. We strongly recommend that these accession numbers appear in any publication which reports or discusses these data. The data are simultaneously made available to other INSDC databases, the European Nucleotide Archive (ENA) and the DNA Data Bank of Japan (DDBJ).

Please reply using the current Subject line.

Sincerely,

Erica Lam
GenBank Direct Submission Staff
gb-admin@ncbi.nlm.nih.gov

GenBank flat file:

```
LOCUS       OQ161123               495 bp    DNA     linear   PRI 01-JAN-2023
DEFINITION  UNVERIFIED: Homo sapiens sequence.
ACCESSION   OQ161123
VERSION     OQ161123
KEYWORDS    UNVERIFIED.
SOURCE      Homo sapiens (human)
  ORGANISM  Homo sapiens
            Eukaryota; Metazoa; Chordata; Craniata; Vertebrata; Euteleostomi;
            Mammalia; Eutheria; Euarchontoglires; Primates; Haplorrhini;
            Catarrhini; Hominidae; Homo.
REFERENCE   1  (bases 1 to 495)
  AUTHORS   Soontornpanawet,C., Singha,K., Srivorakun,H., Tepakhan,W.,
            Fucharoen,G. and Fucharoen,S.
  TITLE     Molecular basis of a high Hb A2 / Hb F beta-thalassemia trait with
            3.4 kb deletion (NG_000007.3:g.69825_73314del3488)
  JOURNAL   Unpublished
REFERENCE   2  (bases 1 to 495)
  AUTHORS   Soontornpanawet,C., Singha,K., Srivorakun,H., Tepakhan,W.,
            Fucharoen,G. and Fucharoen,S.
  TITLE     Direct Submission
  JOURNAL   Submitted (01-JAN-2023) Faculty of Medicine, Mahasarakham
            University, 11/20 Khamriang Sub-District, Kantarawichai, Maha
            Sarakham 44150, Thailand
COMMENT     GenBank staff is unable to verify sequence and/or annotation
            provided by the submitter.
            LocalID: Seq1
            Bankit Comment: BankIt2658994
            Bankit Comment: LocalID:Seq1

            ##Assembly-Data-START##
            Sequencing Technology :: Sanger dideoxy sequencing
            ##Assembly-Data-END##
FEATURES             Location/Qualifiers
     source          1..495
                     /organism="Homo sapiens"
                     /mol_type="genomic DNA"
                     /db_xref="taxon:9606"
BASE COUNT      219 a     77 c     70 g    129 t
ORIGIN
        1 tccccagtta acctcctatt tgacaccact gattacccca ttgatagtca cactttgggt
       61 tgtaagtgac tttttattta tttgtatttt tgactgcatt aagacaaaag aagaaaattc
      121 taatattcac gttgcagccg ttttttgaat ttgatatgag aagcaaaggc aacaaaagga
      181 aaaataaaga agtgaggcta catcaaacta aaaaatttcc acacaaaaaa caaaacaatg
      241 aacaaatgaa aggtgaacca tgaaatggca tatttgcaaa ccaaatattt cttaaatatt
      301 ttggttaata tccaaaatat ataagaaaca cagatgattc aataacaaac aaaaaattaa
      361 aaataggaaa ataaaaaaat taaaaagaag aaaatcctgc catttatggc agaattgatg
      421 aacctggagg atgtaaaact aagaaaaata agcctgacac aaaaagacaa atactacaca
      481 accttgctca tatgt
//
```
